# Molecular Signature in HCV-Positive Lymphomas

**DOI:** 10.1155/2012/623465

**Published:** 2012-08-16

**Authors:** Valli De Re, Laura Caggiari, Marica Garziera, Mariangela De Zorzi, Ombretta Repetto

**Affiliations:** ^1^Unit of Clinical and Experimental Pharmacology, IRCCS, Centro di Riferimento Oncologico, National Cancer Institute, 33081 Aviano, Pordenone, Italy; ^2^Clinical and Experimental Pharmacology, Centro di Riferimento Oncologico, IRCCS, 33081 Aviano, Pordenone, Italy

## Abstract

Hepatitis C virus (HCV) is a positive, single-stranded RNA virus, which has been associated to different subtypes of B-cell non-Hodgkin lymphoma (B-NHL). Cumulative evidence suggests an HCV-related antigen driven process in the B-NHL development. The underlying molecular signature associated to HCV-related B-NHL has to date remained obscure. In this review, we discuss the recent developments in this field with a special mention to different sets of genes whose expression is associated with BCR coupled to Blys signaling which in turn was found to be linked to B-cell maturation stages and NF-**κ**b transcription factor. Even if recent progress on HCV-B-NHL signature has been made, the precise relationship between HCV and lymphoma development and phenotype signature remain to be clarified.

## 1. Introduction

 In the early 2000s, a large body of experimental and epidemiological evidence established an association between B-cell non-Hodgkin lymphoma (B-NHL) and hepatitis C virus (HCV). Epidemiological studies demonstrated that HCV-related type-II mixed cryoglobulinemia herein named (MC), a B-cell lymphoproliferative autoimmune disease, favor lymphoma progression [1]. Approximately 1 of 20 instances out of B-NHLs in Italy may be attributable to HCV [2, 3]. HCV incidence was found to be higher in the south and on the islands [3]. The burden of clinically relevant HCV-positive cases in Italy is on the decline [4]. 

 As of today, the precise mechanism of lymphoma onset remains unclear. HCV has been demonstrated to infect B-cells but the level of replication is low and is only demonstrated in a few cases. The mechanism of B-cell tropism remains elusive, and cell cultures producing HCV are limited [5–7]. Alternatively, though not necessarily in opposition, cumulative evidence supports a role of HCV as an etiological agent for indirect stimulation of specific B-cells, resulting in progressive clonal expansion of B-cells [8–10]. The incidence of cryoglobulinemia and indolent HCV-related B-NHL decreases after HCV eradication, data reinforcing the suggestion of a contribution of chronic antigenic stimulation to the physiopathologic process of HCV B-NHLs [11–13].

 Clinically, HCV has been associated with different histotypes of B-cell B-NHLs which are indistinguishable from typical B-NHL, except for the presence of HCV, the coexistence of liver disease, and the presence of cryoglobulinemia. Because indolent HCV B-NHLs are currently considered a progression of MC related to HCV infection, they are treated in the same way as MC with antiviral therapy (such as pegylated interferon and Ribavirin) [14, 15]. New approaches, such as anti-CD20 monoclonal antibody, have also been proposed alone or in addition to antiviral treatment [11, 16]. HCV-B-NHL has been treated like other lymphomas when symptomatic. 

The strong association between HCV infection and B-NHLs has lead to search for molecular signatures that can predict patients' characteristics, enhance understanding of biological mechanisms of lymphomagenesis, and could have diagnostic/clinical usefulness. 

This paper takes into account gene expression profiling, characterization of B-cell maturation stages, experimental antigen-induced B-cell growth, and immunoglobulin secretion as well as immune-regulatory molecules involved in these processes, which, taken together, provide powerful means to better define HCV-lymphoma entities. Despite these studies, the identification of the molecular signature of HCV-B-NHLs is not completely defined yet and we underscore the need for further studies. 

## 2. HCV + B-NHL Histotypes

 HCV infection has been associated with different histotypes. Splenic marginal zone lymphoma (SMZL) is a rare low-grade B-cell lymphoma (less than 1% of all B-NHLs) but is a commonly found characteristic of HCV infected population, they develop it in about one-third of cases [17, 18]. SMZL displays a strongly homogeneous signature implying the existence of a single molecular entity [19]. Phenotypically, SMZL is usually negative for CD10, CD23, and CD123. They coexpress IgM and IgD, with surface immunoglobulin light chain restriction. Of the genes deregulated in SMZLs, special mention should be made for the genes involved in BCR signaling, tumor necrosis factor signaling, and NF-*κ*B activation [20–22].

 A higher prevalence of HCV positivity was also observed among lymphoplasmacytoid/lymphoplasmacytic/immunocytoma and diffuse large cell histotypes than among HCV-negative counterparts [18]. In primary hepatic lymphomas, mainly of DLBCL type, the prevalence of HCV infection is again higher than that in the HCV-negative population [23].

## 3. B-Cell Receptor 

 It has been previously demonstrated that the B-cell receptor (BCR) repertoire expressed by clonal B-cells involved with HCV-associated MC as well as with B-NHL is not random, with VH1-69 and VH3 heavy chain and VK3-20 and VK3-15 light chain genes being the most represented [9]. These data suggest a model of antigen-driven origin for these lymphoproliferative disorders with the recognition of a limited number of HCV antigens, that is, NS3 [24], E2 [9, 25], and indirectly core-antibody complexes [26, 27]. Moreover, core antigens are proposed as responsible for vascular damage [28] and NS3 antigen as responsible for membranoproliferative glomerulonephritis [29, 30]. 

## 4. Pauciclonality of Peripheral B-cells in Both Resolved and Chronic HCV-Infected Patients

 Pauciclonality of the peripheral B-cell population is a characteristic of HCV-infected patients with MC and/or B-B-NHL [31, 32] and is also a distinguishing feature of subjects who spontaneously resolved HCV infection even though they did not present any clinical manifestation of lymphoproliferative disease [33]. The most important difference between expanded B-cells of resolved and chronically infected patients has been shown in the B-cell CD27− subpopulation. B-cell clones from patients who are spontaneously resolvers preferentially used similar VH, DH, and JH gene segments compared to blood samples from healthy donors, but with a different frequency of the usage of some gene segments with respect to patients with chronically evolving HCV infection (mainly the VH1-69 gene) and higher antigen selection, as shown by the number and characteristics of somatic mutations [33]. CD27 expression has been used to distinguish between memory and naive B-cells in humans; however, low levels of mutated and isotype-switched CD27− cells, typical of a mature B-cell, are also seen in healthy individuals [34]. The atypical enrichment of VH1-69-positive cells in the CD27− B-cell compartment in resolved individuals with respect to chronic HCV-infected patients suggested an accumulation of these “VH-designated” B-cells in these patients [27]. Reported data did not discriminate between the CD27− B-cell subtypes; therefore, it is impossible to distinguish specific B-cell subtype(s) associated with HCV resolvers as of today. 

On the other hand, VH1-69+ cells are associated with mature CD27+ clonal B-cell in HCV-infected patients with MC or B-NHL [31, 35]. Data suggests that MC or B-NHL malignant B-cell clones may develop from VH+ CD27− B-cell subtypes involved in HCV clearance [33].

## 5. Cluster of Differentiation for HCV-B-NHL B-Lymphoid Cells 

 In recent years, it was clearly demonstrated that CD27 is not a universal marker of memory B-cells in human; in fact, its expression distinguishes between different subsets of memory B-cells, and CD27 expression occurs in a distinct developmental fashion of CD27 negative B-cells [34, 36]. Naive and resting B-cells usually do not express CD27, but its expression can be induced by activation of B-cells, resulting in sustained expression over long periods of time. The CDR-H3 repertoire of the CD27− cells is significantly different from the CD27+ cells, indicating that perhaps the lack of a CD27 molecule might be related to binding properties of the immunoglobulin CDR-H3 region [34]. 

 An accumulation of “atypical memory” CD27− B-cells has been described in chronic diseases such as in HIV-infected viremia [37, 38], in plasmodium falciparum-infected patients [39], in individuals from malaria endemic area [40], and in patients with autoimmune diseases such as systemic lupus erythematous (SLE) [39] and rheumatoid arthritis [41]. Therefore, an accumulation of memory CD27− cells is a characteristic feature occurring in several chronically infectious and autoimmune diseases. One hypothesis is that the CD27− memory compartment contains B-cells assigned to produce antibodies to counteract some infections, while at the same time, this compartment also contains autoreactive B-cells. Memory CD27− B-cells hamper the development into antibody-secreting plasma cells through decreased levels of stimulatory molecules and an increased levels of inhibitory molecules expression [34, 36, 39, 42]. Thus, it is suggested that these B-cells become “exhausted” after extensive proliferation driven by microbial antigens. This action should reduce the negative effect of autoreactive antibodies, but by the same mechanism should favor the achievement of a chronic infection due to a reduction in the titer of efficient antibody production. 

With regard to HCV-infection, while HCV resolvers showed an accumulation of VH1-69 CD27− B-cells compared to HCV-chronic patients [33], in MC condition a reduced number of naïve B-cells (CD27−, CD21+, and CD10−) due to an increasing sensitivity to undergo apoptosis was found [43]. Conversely, in MC, Holz et al. evidenced an expansion of both the T2 immature transitional B-cell subset (CD27−, CD21+, and CD10+) and an increase of activated B-cells (CD27+, CD21−, and CD10−) which showed apoptotic resistance [43]. The activated B-cell subset predominantly expresses the VH1-69 segment, suggesting that this B-cell population has to induce the MC disorder [31, 35, 44]. Eradication of HCV with peg-interferon therapy, is associated with a decrease in the number of these last B-cells, but the authors showed that marginal zone-like counterpart (VH1-69, IgM+, CD27+, CD21+, CD11c−), they hypothesized that is was an ancestor of activated B-cells (CD27+, CD21−), may persist after viral eradication and thus should maintain the autoimmunity [45]. Moreover, B-cell depletion using Rituximab treatment for patients who failed antiviral therapy [11] highlights the relationship between clinical MC response and the restoration of T1/T2 transitional immature B-cell ratios by a reduction of the T2 B-cell proliferation, underscoring an important role of this B-cell population in MC pathogenesis [43]. 

 Clonal B-cells from patients with HCV-related B-NHLs had similar mature CD27+ immunophenotype to MC, but in these cases the mechanism of B-cell homeostatic is broken and B-NHLs histotype was heterogeneous.

**Lymphoplasmacytoid**

Lymphoma is a low-grade B-NHL, involving the spleen, bone marrow, and lymph nodes. It produces cryoglobulins and may originate from B-cells that have bypassed the germinal center. Among these B-NHLs, the most typical form associated with HCV infection is the splenic lymphoma. Lymphoplasmacytoid can transform to diffuse large B-cell lymphoma (DLCBL) and originate from memory marginal zone-like B-cells, predominantly IgM+ IgD−, mutated IgVH, CD10−, CD5−, and cyclin D1−. When the neoplastic cells circulate in the peripheral blood they are termed villous lymphocytes due to their characteristic appearance [17].

**DLCBL**

DLCBL is the most common type of aggressive lymphoma with frequent extranodal involvement, and its immunophenotype and genetic features are variable and often aberrant. Primary hepatic DLCBL also shows a high prevalence of HCV infection. Since HCV primarily affects the liver, these data underscore the increase risk of lymphomagenesis following HCV infection in [23].

## 6. Genetic Alterations and BLyS/BAFF Expression in HCV-B-NHLs

 The exact process for altered B-cell homeostasis in HCV-B-NHLs is not yet understood, although accumulation of several genetic anomalies has been highlighted. In particular, trisomy 3q [46] and possibily a higher human telomerase gene (TERC) at 3q23.3 copy numbers were observed in HCV-associated NHL than in HCV-positive patients [47]. Moreover, polymorphisms in oxidative stress genes [48]; deregulation of NF-*κ*B, BCR and TLR pathways in splenic marginal zone lymphomas have also been reported [22, 49]. T(4; 18) translocation involving the Bcl2 gene has also been hypothesized, but this translocation has been found only in some cases, mainly of MALT histotype (lymphoma involving the mucosa-associated lymphoid tissue) [50].

**Figure 1:**
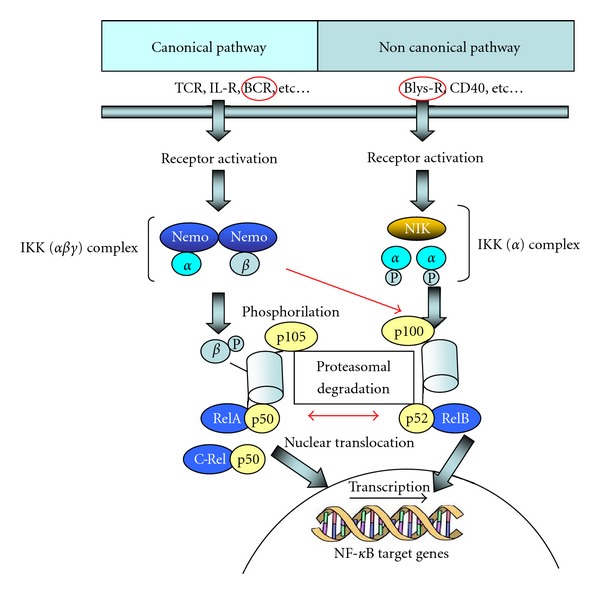
NF-*κ*B members and NF-*κ*B signaling. The NF-*κ*B family is composed of five related transcription factors: p50, p52, RelA (p65), c-Rel, and Rel-B. These transcription factors are related through homology domains in which they form homodimers and heterodimers that bind NF-*κ*B DNA sites, thus modulating gene expression. P50 and p52 are derived from p105 and p100 precursors, respectively. NF-*κ*B is silenced by interactions with inhibitory IkB family members in the cytoplasm. There are two NF-*κ*B signaling pathways known as the canonical pathway (or classical) and the noncanonical (or alternative) pathway. In both pathways, IkB kinase is activated and induces proteasomal degradation of the IkB inhibitor, thus allowing the translocation of the transcription factor subunits into the nucleus and induce transcription of target genes. BCR crosslinking provides the canonical NF-*κ*B signal and p100 production, while BLys receptor induce accumulation of p52, a protein deriving from p100 that activates NF-*κ*B 2 via the noncanonical pathway.

 Several pieces of evidences suggest an important role of B-lymphocyte stimulator factor (BLyS), a TNF family member also known as B-cell activating factor (BAFF) which is expressed in B-NHL and MC [51–53]. The protein is a potent coactivator of immunoglobulin production. Transgenic mice overexpressing BLyS develop B-cell hyperproliferation together with the production of high levels of immunoglobulins like IgM, rheumatoid factor, and cryoglobulins [54]. BLyS typically activates NF-*κ*B, JNK, and ERK pathways that in turn lead to B-cell survival, proliferation, and differentiation (Figure 1). BCR signal generates the canonical NF-*κ*B signal and in some B-cell stages upregulates expression of NF-*κ*B2 genes, resulting in the production of p100 protein [55]. BLys-receptor activation provides an accumulation of p52 protein deriving from p100, which activates NF-*κ*B by the noncanonical pathway [56] (Figure 1). BLyS also plays a role in Ig class switching in mature B-cell differentiation [57]. 

## 7. Maturation of B-cells Is Controlled by BCR/BLyS Interaction

 A strong association between high level of BLyS and cryoglobulinemic syndrome is now clearly confirmed. 

**Figure 2:**
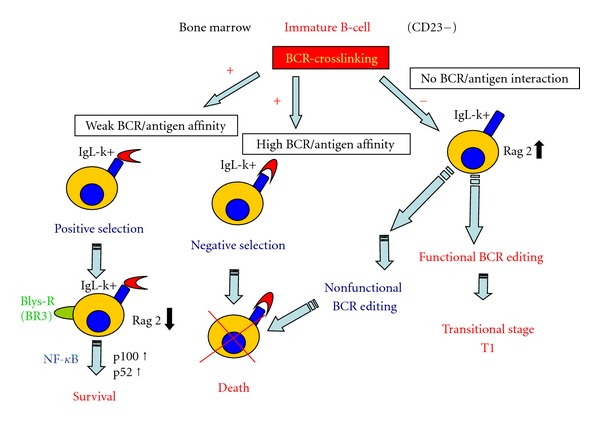
A model of BCR/BLyS interaction of immature B-cells to transitional B-cells. In bone marrow, weak BCR linkage of immature B-cells induces BLyS-receptor expression and a down-expression of RAG-2, an enzyme involved in BCR editing. In addition, immature B-cells can be rescued from the negative selection of the BCR signaling apoptotic pathway. After a functional (but not strong self-reactive) BCR editing is accomplished. In case of high BLyS level, some weak self-reactive B-cells (weak BCR/antigen affinity) can be developed into T-transitional immature B-cell stage.

 High expression levels of BLyS are known to increase the survival of positive selected immature bone marrow B-cells (IgM+, CD23−), regulate peripheral/spleen T2/T3 transitional stages, and improve survival of follicular/splenic mature B-cells [55, 58]. BLyS may induce B-cell proliferation in bone marrow only when acting together with anti-IgM (Figure 2). Several studies also found that BCR ligation upregulated BLyS-receptor expression in transitional T2 and mature B-cells. Conversely, the Fc*γ*IIB receptor, a receptor for the Fc fragment of IgG immunoglobulin, present on B-cells, abolishes the effect by reducing the expression of BLyS-receptor levels [27, 59]. It is hypothesized that alteration in the homeostasis of B-cells development, including autoreactive B-cells, may be caused by excessive levels of BlyS [51, 60]. 

 Recently, it has been suggested that physiologically BLyS steady-state concentrations may be under the feedback control of a number of B-cells present in the individual and BLyS-receptors on various amounts on B-cell surfaces that both bind and then subtract BLyS molecules from the serum [61]. BLys is necessary for B-cell survival and proliferation; therefore, when the number of B-cell decreases, BLys concentration increases and vice versa, thus preserving the stead-stay level of B-cells in the peripheral blood. Moreover, it was found that transitional and naive IgD+ CD27− B-cells require more BLys-induced survival signals than the CD27+ switched memory or marginal zone-like B-cells [62]. This effect has been ascribed to a difference in BLyS receptor family expression in immature, transitional, and antigen-experienced mature B-subsets. BLyS binds three receptors: transmembrane activator and calcium-modulator (BCMA), cyclophilin ligand interactor (TACI), and BR3 (BLyS receptor 3), with decreasing affinity for BLyS in the following order: BR3 > TACI > BCMA [63, 64]. BR3 was first observed in immature B-cell; after antigen-encounters, a shift from BR3 to BR3 plus TACI expression occurs, while BMCA is present only during plasma cell differentiation [64, 65]. BLyS receptor should then regulate B-cell maturation acting on different NF-*κ*B pathways (TACI-receptor activates canonical NF-*κ*B pathway, while BR3 receptor activates NF-*κ*B only through noncanonical signaling (Figure 1)). As a consequence, different subset of B-cell competes for BLyS molecules regulating the quantity of B-cells in each subset. This model of BLyS/B-cell interaction is strongly supported in X-linked agammaglobulinemia patients and in several animal models where mature Blys-receptor+ B-cells were not generated, and where soluble BLys levels were then found higher than that in healthy controls while in other genetically defined primary immunodeficiencies, patients who show switched BlyS receptor positive memory B-cells, have normal BlyS levels [61].

 HCV B-NHLs are almost always transitional/activated IgM+ K+ B-cells [9], a characteristic shared between some immature and mature B-cells which have not yet accomplished immunoglobulin switching. In bone marrow, immature B-cells with BCR receptor reactivity (including self-molecules reactivity) below the threshold induce a negative selection survive and proliferate when BLyS-receptors expression is associated with high IgM expression (Figure 2). Immature transitional B-cells require both a tonic BCR signaling for survival and proliferation [66] and BLyS receptors expression [67], whose expression is in turn regulated by the BCR signal itself [64, 68]. Thus, in immature B-cells, the apoptosis was induced by a strong BCR-crosslinking that generated a BLyS-receptor downregulation, while in transitional mature B-cells, BCR-crosslinking was accompanied by BLyS-receptor upregulation and B-cell proliferation. It is proposed that high levels of serum BLyS may rescue weak self-reactive B-cell clones that usually die at the transitional stage from apoptosis [58]. 

 It has been well documented that in the general population BLys mediated signaling is involved in the survival and proliferation of some B-NHLs [69], with a different effect on BLyS response related to different stage on mature B-cell malignancies [70]. Unlike normal B-cell counterpart, most B-NHLs express BLyS in an autocrine fashion and/or present mutation in the cytoplasmatic region of the BLyS receptor [71]. With regard to HCV-B-NHL, as well as MC, an increase in BLyS levels was found. Although a long-term production of BLyS molecules in response to chronic infections and inflammation cannot be excluded, a BLys autocrine production and/or BLyS receptor mutation should be possible. Studies are required to elucidate this question.

## 8. Immune Response against the VK3-20 Protein and Shift in TH2 Immune Response

 Dendritic cells (DC) are the most powerful cells that process antigen material and present it on the surface of other cells of the immune system; they also play an important role as part of the immune regulatory network. Depending on their lineage (myeloid or plasmacytoid: CD11, CD83, CD86, and HLA-DR class-II markers) and stage of differentiation and activation (CD40 and CD80 markers), dendritic cells may either promote a strong T-lymphocytes-mediated immune response or an anergic state. The frequency of mDC and pDC, along with the expression of CD40 and CD80 markers, indicate the capacity of recombinant VK3 light immunoglobulin to specifically induce DC activation and maturation in healthy subjects as well as in HCV-positive patients [72]. Buonaguro et al. also observed a poor monocyte activation and maturation [72]. In vitro, BCR stimulus of mature peripheral B-cells only (CD19+ and CD27+) yielded a significant increase in expression of activation markers CD80 and CD86, even though the trend for CD86 is not significant on B-cells of HCV-infected patients as compared to healthy control subjects [73]. Moreover, after VK3 stimulation, a higher production of TH2 cytokines (IL-6, IL-4, IL-10, and TNF-*α*) was observed in HCV-positive patients. A shift in TH2 cytokine expression, characterized by an elevated production of some cytokines, had already been reported in other chronic infections such as HIV, Helicobacter pylori and other virus-related diseases [74, 75]. On the other hand, patients with MC have increased levels of TH2 cell-derived cytokines, that is, IL-2 and IL-5 [76]. Therefore, it is suggested that the TH2 immune response, by means of T-cell-dependent B-cell stimulation, may promote autoantibody production, MC disease, and B-NHL. 

 Recent studies propose a crosstalk between TH2 cytokines production and abnormal B-cell activation. The results of these studies indicated an association between HCV and suppressors of cytokine signaling (SOCS), which negatively regulates the cytosol-to-nuclear JAK-STAT signaling which ultimately induces interferon signaling and apoptosis of the cell [77–79]. However, how cytokines might be involved in the development of B-cell clonal expansion, mixed cryoglobulinemia, and B-NHL in patients who are chronically infected with HCV still remains unknown.

## 9. HCV-Positive B-NHL Showed Restricted Combination of HLA Class-II Genes

 Immune complexes may be internalized in B-cell through BCR-ligation, then processed and presented in the context of HLA class-II molecules to T-cell costimulation in the germinal center of follicles [80]. This T/B-cell interaction is necessary for immunoglobulin class switch recombination, somatic hypermutation, and specificity-based selection underlying immunoglobulin affinity maturation. Restricted Ig genes may be presented by means of a limited number of HLA molecules. Possibly because of this, it is evidenced that the DR5-DQ3 HLA combination was strongly associated with the HCV (+) MC (+) B-NHL group of patients compared with bone marrow donor population, while the contribution of DR1-DQ1 was higher in cases of HCV (+) B-NHL without MC [81]. The most common DR-DQ combination class used in HCV-infected patients without lymphoproliferative diseases and in subjects with HCV-related hepatocellular carcinoma was also different [82, 83]. 

## 10. Decreased miRNA26b Expression Associated with HCV-Related Marginal Zone Lymphomas

 MicroRNAs (miRNAs) are a class of small noncoding RNAs that bind to partially complementary sites in the 3′ untranslated regions (UTR) of target mRNAs and modulate gene expression by facilitating translational repression or mRNA degradation. Liver-specific miRNA miR-122 was found to be associated with inhibition of HCV replication. MiR-122 regulates virus production by directly interacting with the 5′ end of the HCV RNA genome [84].

 Only one study, to date, explores miRNA patterns and HCV-related B-NHL [85]. A reduced expression of miRNA-26b has been found in HCV-positive versus HCV-negative patients with SMZL. This latter miRNA seems to be most strongly associated with specific HCV-related MZSLs, since the miRNA pattern is different in chronic HCV-related subjects and in those with HCV-related hepatocarcinoma. One predicted target of miRNA26b is the NIMA-related kinase NEK6, which has a critical role in mitotic cell cycle progression and is upregulated in various human cancers. In the same study, no statistically differential expression between SMZL and nonneoplastic splenic tissue was found. However, a trend towards a significant difference in the expression of 7 out of 381 miRNAs was tested. Notably, from the list of genes reported, the upregulation of miR-21 and miR-155 was associated with several genes involved in NF-*κ*B signaling. Genetic alterations involving the NF-*κ*B pathway were also found in the SMZL subtype of the general population [21, 86]. 

 The overall data reported indicated the importance of NF-*κ*B signaling in lymphoma pathogenesis and the involvement of specific pathways in HCV-infected patients.

## 11. Toll-Like Receptors 2 and 4 and IL-6 Expression and Association with HCV-B-NHL

 The innate immune response involving toll-like receptors (TLRs) has been shown to play an important role in the pathogenesis of many viruses. The continuous interaction between viruses with TLRs may induce a chronic activation of inflammatory cytokine responses that are a risk factor for tumor development. Compared with peripheral blood cells from healthy individuals, TLR4 was found to be upregulated in B-cells after HCV infection [87]. TLR4 expression was found to be mediated by the NS5A HCV protein and may provoke B-cell activation through interleukine 6 production. Moreover, the core HCV-protein may trigger B-cell activation through TLR2 interaction [88]. The production of proinflammatory interleukine 6, which stimulates B-cell activation, has been suggested to contribute to the development of cryoglobulinemia and B-NHL [88]. BCR and TLR signalling pathways were also found to be targeted by genetic changes in SMZL [22]. Moreover, in the general population, genetic variation in TLR1-TLR6 region genes was associated with B-NHL risk, with a specific association for TLR2 variant and MZL [89]. Although all these data are indicative of a functional relation between TLR and HCV-B-NHL, the real relationship between these molecules remains inconclusive.

## 12. Conclusions

 In conclusion, lymphomas that develop in HCV-infected patients seems to combine disease-specific signatures and different sets of genes whose expression is associated with BCR coupled to Blys signaling, which in turn has been linked to B-cell maturation stages and to specific NF-*κ*B transcription factors. This paper highlights the close link between the specific contribution of these genes in comparison to normal and to chronic HCV-infected B-cells. This paper underscores that HCV-related lymphomas are subject to specific deregulation induced by the virus infection, although the precise relationship between HCV and lymphoma development and phenotype signature needs to be clarified. Identification of molecular signatures in lymphomas occurring in the HCV-infected population could facilitate a more rational approach to the diagnosis as well as more tailored treatments and/or prevention.
